# Proliferating Cell Nuclear Antigen (PCNA) Regulates Primordial Follicle Assembly by Promoting Apoptosis of Oocytes in Fetal and Neonatal Mouse Ovaries

**DOI:** 10.1371/journal.pone.0016046

**Published:** 2011-01-06

**Authors:** Bo Xu, Juan Hua, Yuanwei Zhang, Xiaohua Jiang, Huan Zhang, Tieliang Ma, Wei Zheng, Rui Sun, Wei Shen, Jiahao Sha, Howard J. Cooke, Qinghua Shi

**Affiliations:** 1 Hefei National Laboratory for Physical Sciences at Microscale, School of Life Sciences, University of Science and Technology of China, Hefei, China; 2 Department of Animal and Poultry Science, University of Guelph, Ontario, Canada; 3 Laboratory of Reproductive Medicine, Department of Histology and Embryology, Nanjing Medical University, Nanjing, China; 4 MRC Human Genetics Unit and Institute of Genetics and Molecular Medicine, Western General Hospital, Edinburgh, United Kingdom; Institute of Zoology, Chinese Academy of Sciences, China

## Abstract

Primordial follicles, providing all the oocytes available to a female throughout her reproductive life, assemble in perinatal ovaries with individual oocytes surrounded by granulosa cells. In mammals including the mouse, most oocytes die by apoptosis during primordial follicle assembly, but factors that regulate oocyte death remain largely unknown. Proliferating cell nuclear antigen (PCNA), a key regulator in many essential cellular processes, was shown to be differentially expressed during these processes in mouse ovaries using 2D-PAGE and MALDI-TOF/TOF methodology. A V-shaped expression pattern of PCNA in both oocytes and somatic cells was observed during the development of fetal and neonatal mouse ovaries, decreasing from 13.5 to 18.5 dpc and increasing from 18.5 dpc to 5 dpp. This was closely correlated with the meiotic prophase I progression from pre-leptotene to pachytene and from pachytene to diplotene when primordial follicles started to assemble. Inhibition of the increase of PCNA expression by RNA interference in cultured 18.5 dpc mouse ovaries strikingly reduced the apoptosis of oocytes, accompanied by down-regulation of known pro-apoptotic genes, e.g. Bax, caspase-3, and TNFα and TNFR2, and up-regulation of Bcl-2, a known anti-apoptotic gene. Moreover, reduced expression of PCNA was observed to significantly increase primordial follicle assembly, but these primordial follicles contained fewer guanulosa cells. Similar results were obtained after down-regulation by RNA interference of Ing1b, a PCNA-binding protein in the UV-induced apoptosis regulation. Thus, our results demonstrate that PCNA regulates primordial follicle assembly by promoting apoptosis of oocytes in fetal and neonatal mouse ovaries.

## Introduction

Development of germ cells in female rodents initiates with the migration and colonization of primordial germ cells (PGCs) from the yolk sac to the urogenital ridges [1]. Once the PGCs reach the gonadal anlagen at about 8.5 days post-coital (dpc), the PGCs proliferate continuously until they enter meiosis, after which the cells are referred to as oocytes [2], [3]. Oocytes undergo first meiotic prophase and arrest at diplotene at approximately 17.5 dpc in the mouse until ovulation [4], [5].

Primordial follicles are crucial for fertility of mammalian females throughout their entire reproductive life [5], [6], [7] and are formed in neonatal mouse ovaries. During primordial follicle formation, large cysts (a special cluster of oocytes) have been proposed to break into smaller cysts, and this process is repeated until a few individual oocytes remain [7]. Some of the individual oocytes are finally packaged into primordial follicles, and two-thirds of oocytes die in this process [5], [7]. Several possible mechanisms, including apoptosis [7], [8], [9], [10], [11], autophagic cell death [10], [11], [12], and oocyte extrusion from ovaries [12], have been proposed for oocyte loss, with apoptosis being the major mechanism revealed by almost all *in vivo* and *in vitro* studies by different groups [7], [8], [9], [13], [14]. This has been corroborated by observations in mouse models after deletion of apoptosis-regulating genes [13], [14]. Autophagic oocyte death was observed by two groups under specific culture conditions, where individual isolated oocytes but not intact ovaries were cultured *in vitro* [10], [11], or newborn mouse ovaries were cultured in the absence of serum [12]. Recently, based on analysis of static images of mouse ovaries, Rodrigues et al. [12] suggested that oocyte extrusion from cultured ovaries could result in loss of a significant fraction of oocytes during primordial follicle formation, however, current technologies can not definitively elucidate of this process.

To understand which proteins regulate promordial follicle formation, we used 2D-PAGE and MALDI-TOF/TOF to identify proteins differentially expressed around the time of primordial follicle formation. Proliferating cell nuclear antigen (PCNA) is one of the differentially expressed proteins identified. PCNA is a 36 KDa protein which is well-conserved in all eukaryotic species from yeast to human. The expression of PCNA has been reported in fetal and adult ovaries in several arthropods and mammals, but with considerable variation [15], [16], [17], [18], [19], [20], [21], [22]. In *Marsupenaeus japonicus*, the expression of PCNA mRNA changes significantly with the development of the ovaries [15]. PCNA expression is found in oogonia and oocytes in meiosis I prophase, and increases during oocyte growth in zebrafish [19]. In rabbit ovaries, only part of the oocytes in cysts and primordial follicles are PCNA-positive, and most oocytes stained positive for PCNA in developmental follicles [21]. In adult pig, bovine, and baboon ovaries, PCNA staining was absent from granulosa cells and oocytes of the quiescent primordial follicles, but was intensive in most granulosa cells and oocytes in primary-to-large antral follicles [23], [24], [25]. Oktay *et al.* reported that in rat ovaries, the expression of PCNA was not detected in granulosa cells or oocytes in primordial follicles, but increased with the initiation of follicle growth [17]. In two recent papers, however, oocytes in all stages of follicles, including primordial follicles, were labeled by PCNA antibody in adult rat ovaries, and PCNA staining was suggested as a marker for ovarian follicle counts [16], [18]. Recently, the distinct expression of PCNA was reported with the development of fetal and newborn rat ovaries, with PCNA-positive oocytes observed at high percentages from 14.5 dpc to 1 day post-partum (dpp), decreasing after birth, and increasing during primordial follicle formation [22]. In mouse ovaries, a complicated PCNA expression pattern was reported in a recent study, in which PCNA staining was intensive in oocytes at 1 dpp when primordial follicles actively assembly, absent in oocytes in all follicles from 14–25 dpp, and absent in primordial, but not primary or secondary follicles from 7–12 dpp and 50–200 dpp [20]. Thus, in mammals the expression of PCNA in oocytes of various meiotic I prophase before primordial follicle formation and in primordial follicles is incompletely understood.

Distinct expression usually suggests an important role of interested proteins during biological processes, and it has been shown that PCNA serves as a key factor in many essential cellular processes, such as DNA replication, DNA repair, sister-chromatid cohesion, DNA damage avoidance, cell cycle control, and cell survival [26], [27], [28], [29]. PCNA has also been suggested to be a key regulator during the development of ovarian follicles [30], but the exact function of PCNA in meiosis, particularly in primordial follicle formation, remains unknown.

In mammals, functions of a protein/gene in the development of germ cells and ovaries are usually studied by gene manipulation [31], [32], antibody neutralization [33], [34], or chemical treatment [35], [36]. However, targeted deletion of PCNA in mice resulted in embryonic death before the initiation of meiosis [37]; antibodies supplied in culture medium may not neutralize PCNA activity in ovaries because of the localization to the nucleus of this protein, and chemicals specifically inhibiting PCNA are not available. Fortunately, Wang and Roy successfully suppressed gene expression in cultured newborn hamster ovaries using RNA interference (RNAi), which provides a convenient and powerful strategy to explore functions of an interested gene during female meiosis and primordial follicle formation [38].

In the present study, we detected expression of PCNA in fetal and neonatal mouse ovaries during meiotic prophase progression and primordial follicle formation, and explored the possible roles played by this protein in primordial follicle formation using a modified RNAi approach [38]. A V-shaped expression of PCNA in both oocytes and somatic cells was observed in ovaries from 13.5–18.5 dpc and from 18.5 dpc to 5 dpp, which is closely correlated with the meiotic prophase I progression from pre-leptotene to pachytene and from pachytene to diplotene. Inhibition of the increased expression of PCNA in ovaries around primordial follicle formation alleviates apoptosis of oocytes and increases primordial follicle assembly. Thus, this report provides strong evidence that PCNA plays a crucial role in oocyte loss during ovarian follicle formation.

## Materials and Methods

### Animals

Male and female ICR mice, which have been used widely for reproduction research [39], [40], were purchased from the National Rodent Laboratory Animal Center (Shanghai Branch, China). All mice were maintained according to the Institutional Animal Care and Use Committee of University of Science and Technology of China. Fetal or neonatal mouse ovaries were obtained from the pregnant mice between 13.5–19.5 dpc (0 dpp) and 1–5 dpp. Noon of the day when the copulatory plug was found was designated as 0.5 dpc or 0 dpp.

### 2D-PAGE, MALDI-TOF/TOF and identification of PCNA

To identify proteins differentially expressed in 13.5 dpc, 16.5 dpc, and 1 dpp mouse ovaries, 2D-PAGE, MALDI-TOF/TOF, and Statistical analysis was carried out as described [41]. Differentially expressed proteins were identified by the mass chromatographic/mass chromatographic spectra, and results of mass were cross-referenced with the International Protein Index mouse database (http://www.ebi.ac.uk/IPI/IPIhelp.html). Only the database entry exhibiting the highest number of match in peptides was included.

### Immunohistochemistry

Ovaries or testes were fixed in 10% formaldehyde for 12–24 hours before paraffin embedding, and 5 µm paraffin sections were attached to microscope slides. Paraffin sections were heated at 60°C for 2 hours. Following de-paraffinization, sections were re-hydrated in a series of graded ethanol/water solutions, then boiled in 10 mM citric acid (pH 6.0) at 95–100°C for 10 minutes followed by incubation in 3% hydrogen peroxide (H_2_O_2_) for 10 minutes. The tissues were blocked by BDT (3% BSA, 10% normal donkey serum in TBS) and incubated with a mouse anti-PCNA monoclonal antibody (Zhong Shan Goldenbridge Biotechnology, Beijing, China; 1∶100), or rabbit anti-MVH antibody (A kindly gift from Professor Toshiaki Noce, Mitsubishi Kagaku of life Sciences, Japan; 1∶1000) or a mouse anti-Ki-67 monoclonal antibody (Zhong Shan Goldenbridge Biotechnology, Beijing, China; 1∶100) overnight at 4°C. After rinsing thoroughly with TBS, the sections were incubated with a biotinylated and streptomycin-labeled goat anti-mouse antibody (Maixin Bio, KIT-5010, Fujian, China) for 15 minutes at room temperature or an Alexa 488-conjugated donkey anti-rabbit gamma globulin antibody (Molecular Probes, A21206, CA, USA; 1∶250) for 1 hour at 37°C. PCNA expression in sections was detected by the reaction of peroxidase with 3,3′-diaminobenzidine tetrahydrochloride (DAB) and analyzed using a Olympus BX61 fluorescence microscope.

### Western blot analysis

Lysates from ovaries *in vivo* or cultured *in vitro* were separated on 15% SDS polyacrylamide gels and the proteins were then transferred to nitrocellulose membranes (Amersham Biosciences, RPR303D). The membranes were blocked in TBST (0.5% Tween-20 in TBS) containing 5% nonfat milk powder for 1 hour, incubated overnight with a mouse anti-PCNA monoclonal antibody (1∶100), a mouse anti-beta actin monoclonal antibody (Abcam Ab52, Cambridge, UK; 1∶1000), a rabbit anti-Becn1 polyclonal antibody (Santa Curz, SC11427, CA, USA; 1∶200), a mouse anti-Atg5 monoclonal antibody (A kindly gift from Professor Hans-Uwe Simon, University of Bern, Switzerland; 1∶500) or a rabbit anti-LC3 (Novus Biological, NB100-2220, CO, USA; 1∶1000) polyclonal antibody in TBST, then washed three times (10 minutes each) with TBST. The membranes were then incubated for 1 hour with alkaline phosphatase (AP)-conjugated anti-mouse IgG (Promega, S372B, WI, USA; 1∶1000). PCNA and beta-actin protein levels were evaluated by the detection of activity of alkaline phosphatase using a Lumi-Phos kit (Pierce Biotechnology, KJ1243353).

### Meiotic prophase I oocyte preparation

Meiotic prophase I oocyte spreads were prepared using cytospinning. Briefly, ovaries were shredded, and the released cells were spun onto slides at 800 rpm for 2 minutes by cytospin (Ttettich, MIKRO22R, Tullingen, Germany). Then the slides were incubated with 1% paraformaldehyde solution at PH 7.4 (Sangon, Shanghai, China) for 5 minutes and incubated in 70% ethanol for 10 minutes. The tissues were blocked by BDT with 0.2% Triton-X (Sangon) for 15 minutes and incubated with mouse anti-PCNA monoclonal antibody (1∶100) and rabbit anti-SCP3 (A kindly gift from Professor Peter Moens, Department of Biology, York University, Canada; 1∶200) overnight at 37°C. These primary antibodies were detected by incubation at 37°C for 90 minutes with the following secondary antibodies: Alexa 555 donkey anti-mouse (Molecular Probes, A21432; 1∶250) and Alexa 488 donkey anti-rabbit (Molecular Probes, A21206; 1∶250). At last 10 µl of vectashield mounting medium (Vector Laboratories, H-1000, CA, USA) was applied per slide, and a cover slip was sealed in place.

### Culture of fetal mouse ovaries

The ovaries from 18.5 dpc mice were cultured as described [42]. Briefly, the ovaries without attached mesonephroses were placed on Millicell-PC membrane inserts (Millipore, PIHT12R48, MA, USA) covered with only a thin film of culture medium, and on each membrane only one ovary was placed. The medium for organ culture contained DMEM-F12 (HyClone, SH40007-13, UT, USA)/a-MEM (HyClone, SH30256.01B) (1∶1) supplemented with 0.23 mM pyruvic acid (Gibco, 11360-070, CA, USA), 3% (w/v) BSA (Sigma, A-7906, MO, USA) and insulin-transferrin-selenium mix (Gibco, 41400-045), and did not contain any antibiotics. The organ cultures were maintained at 37°C and 5% CO_2_ in modular incubation chambers (Thermo, 3111, MA, USA). The medium was changed every 48 hours after culture initiation with replacement of half of the complete medium.

### RNAi on cultured fetal mouse ovaries

Ovary RNAi was initiated as described [38]. Cultured ovaries were transfected with 100 nM siRNAs (Invitrogen, CA, USA) using Metafectene (Biontex, T020-5.0, Munich, Germany) following supplier's instructions. The medium was changed at the 48th hour after RNAi initiation with replacement of half of the complete medium without siRNAs.

To determine the transfection efficiency of siRNAs, ovaries were transfected with100 nM Cyanine 3 (Cy3) labeled siRNAs targeting luciferase GL2 (Dharmacon, D-1001110-01-20, TX, USA). Ovaries were harvested after transfection for 96 hours, and cryostat sectioned at 10 µm or dispersed into single cells mechanically. Single ovarian cells were dropped onto microscope slides. Cy3-labeled cells in cryostat sections and on microscope slides were quantified using a BX61 Olympus fluorescence microscope.

To determine if interferon-like response was invoked by siRNA transfection in cultured ovaries, real-time PCR was used to measure mRNA content of Oas1, a classical interferon target gene [43], [44], in ovaries transfected with siRNAs targeting PCNA or luciferase GL2 mRNAs.

The sequence of PCNA siRNAs is 5′-GGCATTGCTAGAAATTGAGAA-3′ targeting 930–950 nt of PCNA mRNA [45], [46], and the nontargeting control siRNAs sequence is 5′-TTCTCCGAACGTGTCACG-3′ which has no homology to any known mouse mRNAs. These siRNAs were chemically synthesized by Invitrogen.

### Proliferation and apoptosis analysis

To assess proliferation, 18.5 dpc mouse ovaries were cultured in the medium with 100 µM bromodeoxyuridine (BrdU) (Sigma, B9285) added 24 hours before harvesting ovaries [47]. Only the cells undergoing DNA synthesis could incorporate BrdU. The ovaries were fixed and embedded in paraffin and sectioned at 5 µm. BrdU-labeled cells in the sections were detected immunohistochemically using a mouse anti-BrdU monoclonal antibody (Molecular Probes, A21300; 1∶100) and imaged using a BX61 Olympus fluorescence microscope. Apoptotic ovarian cells were detected in sections by the TUNEL assay according to the manufacturer's specifications. Ovarian sections were imaged and TUNEL assay positive cells were counted manually using Image-Pro plus software.

### Isolation of oocytes and somatic cells, RNA isolation, RT-PCR, and real-time PCR

Oocytes and ovarian somatic cells were isolated from the cultured ovaries using EDTA-Stab method reported by De Felici and McLaren and a method by O W-S and Baker TG, respectively [48]. The purity of the isolated germ cells and somatic cells was assayed by detecting the mRNA level of Cyp19, a specific somatic cell marker and Figα, a specific oocyte marker [49], [50]. Ovaries and oocytes were collected in RNase-free tubes and total RNAs were extracted using Trizol reagents (Invitrogen, 15596018). cDNAs were synthesized using the prime Script^TM^ 1^st^ strand cDNA synthesis Kit (Takara, D6110A, Osaka, Japan) according to the manufacturer's protocol. Briefly, ovaries were ground sufficiently using an RNase-free grinding rod in Trizol reagent, then methyl chloroform was added to the 1/5 volume of Trizol and mixed thoroughly. The mixture was centrifugated at 14,000 g (4°C) for 15 minutes. The supernatants were transferred to new RNA-free tubes and mixed with isopropyl ketone at a ratio of 1∶1, then centrifuged at 14,000 g (4°C) for 10 minutes after incubation for 10 minutes. The supernatants were removed, 100 µl 75% ethanol (diluted in DEPC water) was added to the tubes and mixed gently. After centrifugation at 7,500 g (4°C) for 5 minutes, the supernatant was removed and the tubes were air-dried at room temperature. To synthesize cDNAs, 2 µl Oligo dT primer (50 µM), 2 µl dNTP mixture (10 mM) and 5 µl RNase-free H_2_O were added to each tube, and the tubes were heated at 65°C for 5 minutes, then snap-cooled on ice immediately. Four µl 5xPrimeScript^TM^ buffer, 0.5 µl RNase inhibitor (40 U/µl), 1 µl PrimeScript^TM^RTase (200 U/µl) and 4.5 µl RNase-free H_2_O were added to each tube, and heated for 1 hour at 42°C for cDNA extension followed by inactivation of the enzymes at 70°C for 15 minutes. The mixture was then frozen at −30°C until used. Real-time quantification of mRNAs was done using a SYBR premix Ex Taq^TM^ II kit (Takara, DRR081A) according to the manufacturer's protocols. Briefly, real-time PCRs were performed in a total volume of 10 µl, and each reaction contained 5 µl SYBR PREmis EX Taq^TM^ (2x), 0.4 µl forward and reverse PCR primers (10 µM), 0.2 µl ROX reference Dye II, 1 µl cDNA and 3 µl ddH_2_O. The PCR program consisted of an initial step of 10 seconds at 95°C, followed by 40 cycles of denaturing at 95°C for 5 seconds and extension at 60°C for 31 seconds. All PCR primers used were listed in Table S1.

### Morphometric evaluation of oogenesis and folliculogenesis

To identify oocytes and follicles, ovarian sections were immunohistochemically labeled with an antibody recognized MVH, a protein specifically expressed in germ cells [51], and Hoechst 33342 for nuclei of ovarian cells. The sections were analyzed using an Olympus fluorescence microscope. All cells labeled by MVH antibodies (oocytes) were counted, no matter whether they were enclosed by granulosa cells or not. Based on their localization, oocytes were designed as naked oocytes, or in large cysts, small cysts, primordial or primary follicles according to previous reports [7], [52]. Germline cysts are clusters of interconnected germline cells. Based on the number of oocytes in each cluster, cysts were classed as small cysts if they had 2–4 oocytes, and large cysts if they contained more than 4 oocytes. Naked oocytes were those that did not locate in cysts and had 0–1 granulosa cells at the surroundings. The oocytes enclosed by at least two squamous granulosa cells were termed primordial follicles. Based on the number of enclosing granulosa cells, primordial follicles were further divided into three categories: type1, the oocytes were enclosed by 2–3 granulosa cells, type 2, by 4–5, and type 3, by more than 5. Oocytes surrounded primarily by a single layer of cuboidal granulosa cells were designated in primary follicles.

### Statistical analysis

In each experiment group, 4–6 ovaries were used, and all experiments were repeated 2–5 times. For each ovary, oocytes, somatic cells or follicles were counted in at least 3 randomly chosen ovarian sections. To eliminate errors in oocyte identification and counting, all analyses were performed in a double-blind manner by two or three individuals and the data were pooled. For real-time PCR and Western blot analysis, two or three cultured ovaries per group were pooled to form one sample, and repeated at least three times for statistical analysis. Chi-square Goodness-of-Fit test was used for correlation analysis. All comparisons between two groups were performed using the paired student's t-test except quantitative data of real-time PCR were analyzed using unpaired t test or one-way ANOVA with unpaired student's t-test. P<0.05 was considered significant.

## Results

### PCNA expression varies during the development of fetal and neonatal mouse ovaries

**Figure 1:**
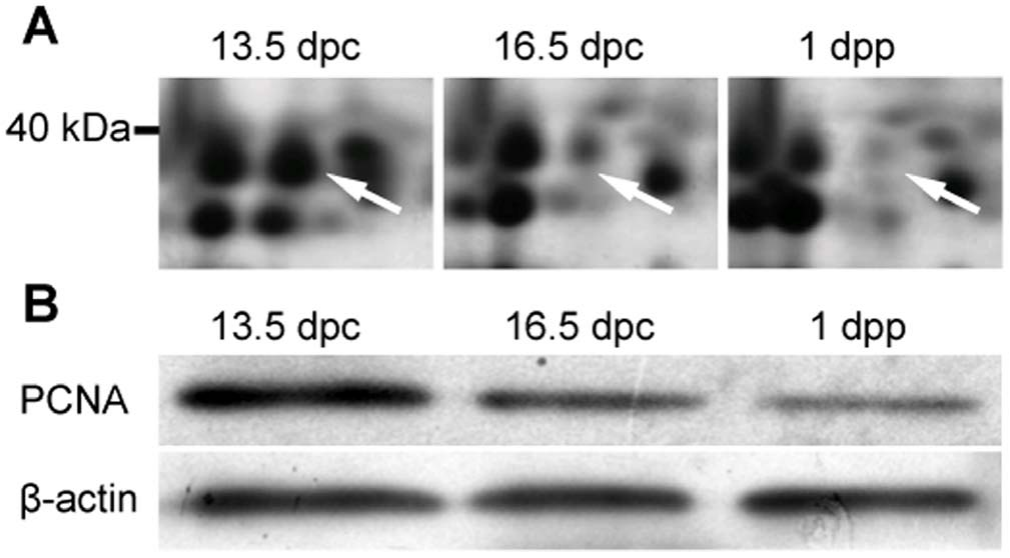
Differential expression of PCNA protein in fetal mouse ovaries. (**A**) Expression of PCNA (arrow) in fetal and neonatal mouse ovaries decreased from 13.5 dpc to 1 dpp detected by 2D-PAGE. (**B**) The decreasing expression of PCNA in mouse fetal ovaries from 13.5 dpc to 1 dpp was confirmed by Western blot analysis using a monoclonal antibody against mouse PCNA.

In fetal mouse ovaries, meiosis initiates at 13.5 dpc, progresses to pachytene of meiosis I at 16.5 dpc in most oocytes, and reaches and arrests at diplotene at 1 dpp in almost all oocytes [5], [7]. Primordial follicle assembly starts at 17.5 dpc and continues during the following 4–5 days, with approximately 30% of oocytes enclosed in primordial follicles at 1 dpp (data not shown). To understand which proteins regulate the progression of meiotic prophase I and primordial follicle formation, 2D-PAGE and MALDI-TOF/TOF technology were used to identify proteins differentially expressed in 13.5 dpc, 16.5 dpc, and 1 dpp fetal or neonatal mouse ovaries. PCNA is one of the differentially expressed proteins identified. PCNA is highly expressed in 13.5 dpc ovaries, and significantly decreased with the development of fetal ovaries (Fig. 1A). This finding was confirmed by Western blot analysis using an anti-PCNA monoclonal antibody (Fig. 1B).

**Figure 2:**
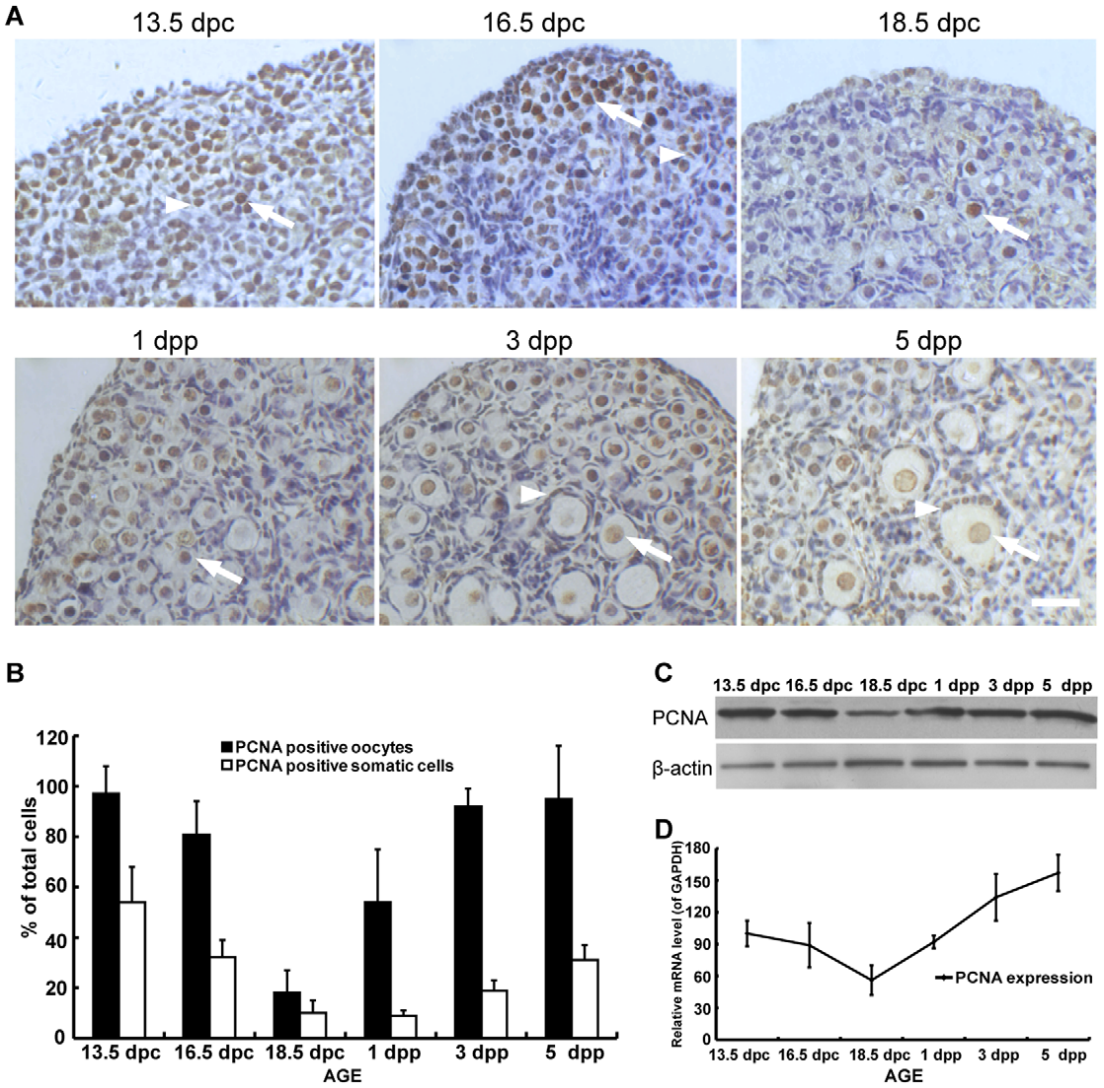
PCNA protein and mRNA level varies with the development of mouse ovaries. (**A**) The expression and localization of PCNA protein in fetal and neonatal mouse ovaries was detected immunohistochemically using a monoclonal antibody against mouse PCNA in ovary sections (Arrow: PCNA positive oocytes; Arrowhead: PCNA positive somatic cells). Bar: 25 µm. (**B**) Quantification of oocytes or somatic cells labeled by PCNA antibodies in mouse ovaries. The expression of PCNA protein (**C**) and mRNA (**D**) varied in fetal and neonatal mouse ovaries detected by Western blot and real-time PCR. Each bar represents a mean±s.d. of 24 (B) and 12 (D) ovaries from two or three independent experiments in different animals.

To more thoroughly understand the pattern of expression and to determine the cell localization of PCNA protein during the development of fetal and neonatal ovaries, an immunohistochemistry technique was applied to sections of 13.5 dpc, 16.5 dpc, 18.5 dpc, 1 dpp, 3 dpp, and 5 dpp ovaries. PCNA protein was detected in both oocytes (round) and somatic cells (fusiform or cube) in all the ovaries examined (Fig. 2A). However, the number of oocytes and somatic cells expressing PCNA varied distinctly in ovaries of various developmental stages (Fig. 2B). In 13.5 dpc ovaries, almost all of the oocytes (97%) were labeled by PCNA antibody. This number decreased with the development of ovaries and reached the lowest in 18.5 dpc ovaries, in which 18% of oocytes stained positive for PCNA. Then, the PCNA-positive oocytes increased rapidly to 54% in 1 dpp ovaries and reached a plateau in 3 dpp ovaries (>92%), at which time primordial follicles start growing. For somatic cells expressing PCNA, a similar pattern, but much fewer cells, were observed in ovaries of different developmental stages examined when compared to PCNA-positive oocytes (Fig. 2B). These observations were consistent with the expression of PCNA protein detected by Western blot analysis (Fig. 2C), and corroborated the findings on PCNA mRNA levels during ovary development (Fig. 2D).

**Figure 3:**
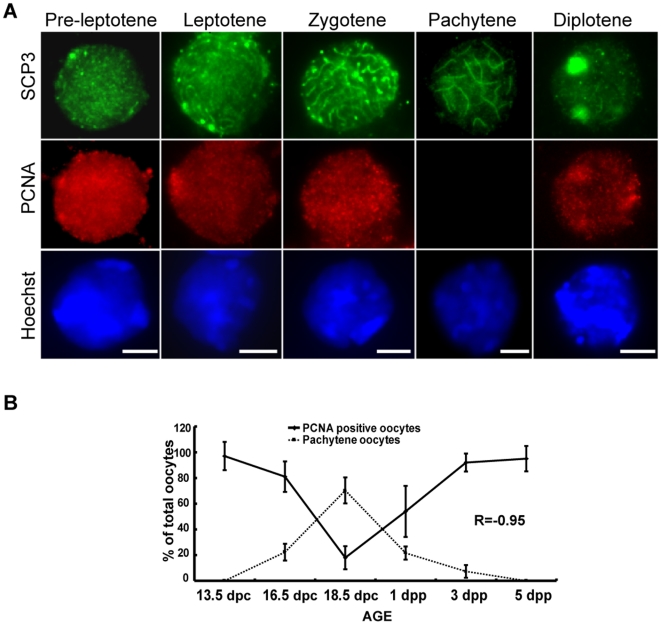
The expression of PCNA is associated with the development of oocytes in fetal mouse ovaries. (**A**) Examples of oocytes at every sub-stages of meiotic prophase prepared by cytospinning from fetal mouse ovaries and immunostained for SCP3 (Green) and PCNA (Red). Bar: 10 µm. (**B**) The percentage of PCNA positive oocytes was negatively correlated with the frequency of pachytene oocytes during the development of fetal and neonatal mouse ovaries. Each bar represents a mean±s.d. of data from 24 or 16 ovaries from two independent experiments in different animals. R = −0.95, Chi-Square Goodness-of-Fit test.

To investigate the correlation between PCNA expression and development of germ cells in meiotic prophase I, double-staining was performed using SCP3 and PCNA antibodies. Based on the localization of SCP3 in microspreading preparations of oocytes, the different stages of meiotic prophase I could be easily distinguished [53], unfortunately PCNA could not be detected by immunostaining in these preparations (probably due to the extraction of PCNA protein from the nuclei). To preserve nuclear PCNA, oocytes were cytospun onto microscope slides for SCP3 and PCNA staining. The oocytes in pre-leptotene, leptotene, zygotene, and diplotene, but not in pachytene, were observed to be labeled by PCNA antibody (Figs. 3A, B). Similarly, we noted PCNA staining of spermatogonia and leptotene spermatocytes, but not pachytene spermatocytes in adult mouse testis sections (Fig. S1A), which is consistent with a previous report [54]. Thus, our results indicated rigorous dynamic expression of PCNA in oocytes during meiotic prophase I progression and primordial follicle formation.

### Liposome-mediated delivery of siRNAs into cultured fetal mouse ovarian cells down-regulates PCNA expression efficiently and specifically

**Figure 4:**
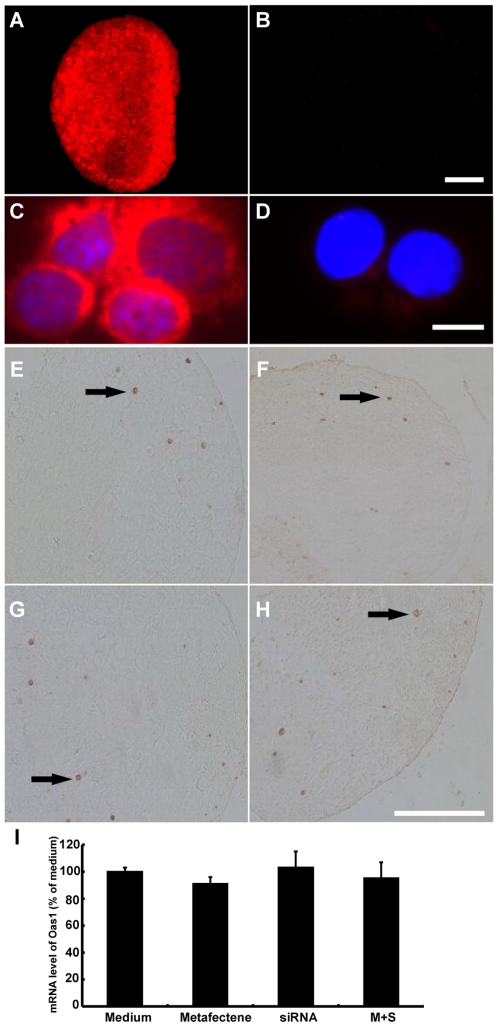
Validation of RNAi system in cultured fetal mouse ovaries. (**A–D**) Efficiency of delivery siRNAs into ovarian cells was monitored by transfecting cultured mouse ovaries with Cy3-labeled nontargeting siRNAs. Ovaries from 18.5 dpc fetal mice were cultured for 96 hours in the presence (A, C) or absence (B, D) of transfection reagent metafectene (2 ul/ml) and Cy3-labeled siRNAs against luciferase GL2 at 100 nM. The Cy3 fluorescence was detected either in cryostat sections (A and B) or single cells of ovaries (C and D) after 96 hours of transfection. Single ovarian cells were obtained by mechanically dispersing the ovaries and depositing on microscope slides by centrifugation for fluorescence detection. Bar: (B) 100 µm and (D) 10 µm. (**E–H**)The toxicity of RNAi reagents was determined by the inspection of morphology of ovaries receiving various treatments, and counting apoptotic cells labeled by TUNEL in treated ovaries. Sections from 18.5 dpc ovaries cultured for 96 hours in vitro (E) in medium alone, (F) medium with metafectene, (G) medium with nontargeting siRNAs, and (H) medium with nontargeting siRNAs and metafectene. (Arrow: apoptotic oocytes). Bar: 100 µm. (**I**) mRNA expression of Oas1, a classical interferon target gene, was measured to determine interferon response by real-time PCR in ovaries receiving various treatments. Each bar represents a mean±s.d. of 16 ovaries from three independent experiments in different animals.

The RNAi strategy has been widely and intensively used in gene/protein function analysis in various systems, *in vivo* and *in vitro* [55], and recently in cultured hamster ovaries [38], but it has rarely been applied to the studies of gene/protein function in cultured mouse ovaries. It is thus necessary to validate the RNAi technology in cultured mouse ovaries. We first examined the efficiency of delivery of siRNAs to cultured mouse ovaries by using Cy3-labeled non-targeting siRNAs as an indicator. Ninety-six hours following metafectene-mediated transfection of cultured 18.5 dpc mouse ovaries with Cy3-labeled non-targeting siRNAs, bright Cy3 fluorescence was observed in almost all of oocytes and somatic cells in cryostat sections (Figs. 4A, B). This is confirmed by the observation of single cells from ovaries treated with Cy3-labeled siRNAs, in which 92% of oocytes and somatic cells showed intense Cy3 fluorescence (Figs. 4C, D). These data showed that siRNAs could be delivered efficiently into ovarian cells and sustained at a considerable concentration in the cells throughout the culture period under our experimental conditions.

**Figure 5:**
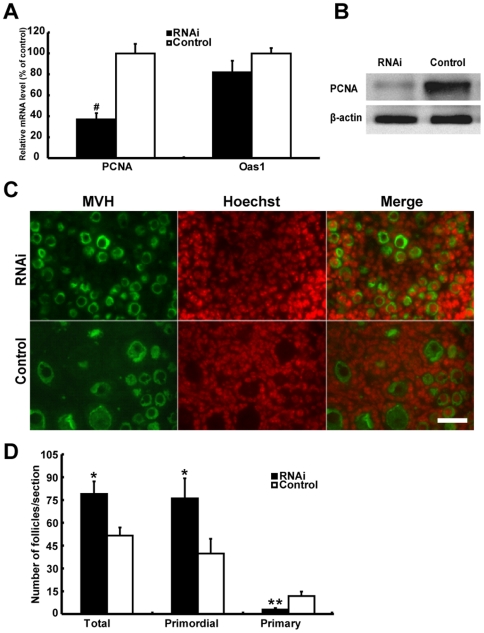
PCNA RNAi effciency and specificity is validated in cultured fetal mouse ovaries, and down-regulation of PCNA in fetal mouse ovaries increases the number of primordial follicles. (**A**) PCNA and Oas1 mRNA levels were determined by real-time PCR in 18.5 dpc ovaries transfected with nontargeting siRNAs (Control) or siRNAs against PCNA for 96 hours. (**B**) PCNA protein levels were determined by Western blot in 18.5 dpc ovaries transfected for 96 hours. A representative image of four independent experiments is shown. (**C**) Follicles/oocytes were detected immunohistochemically in sections of PCNA RNAi or control ovaries. MVH is a protein that specifically expresses in the cytoplasm of germ cells. Bar: 25 µm. (**D**) Quantification of primordial and primary follicles in PCNA RNAi and control ovaries from five independent experiments in different animals. Each bar represents a mean±s.d. of 16 (A) and 20 (D) ovaries. #: P<0.05, unpaired t test, *: P<0.05, **: P<0.01, student's t-test.

Another major concern of RNAi technology is toxicity and interferon response invoked by RNAi reagents [56]. We tested the toxicity of the RNAi reagents on cultured fetal mouse ovaries by inspection of ovarian morphology and the fraction of apoptotic cells indicated by TUNEL labeling. After a 96-hour treatment on 18.5 dpc ovaries with either transfection reagent (metafectene) alone, non-targeting siRNAs alone, or both together following our transfection protocols, the morphology and size of ovaries and the number of apoptotic cells in sections were indistinguishable from the untreated controls (Figs. 4E–H). To evaluate the interferon response after transfection, the mRNA level of 2′-5′ oligoadenylate syhthase1 (Oas1), a classical interferon target gene [43], [44], was measured by real-time PCR. A similar Oas1 mRNA level was observed in ovaries treated or untreated with metafectene, non-targeting siRNAs, or both (Fig. 4I). More importantly, after a 96-hour transfection of 18.5 dpc ovaries with siRNAs targeting PCNA, PCNA mRNAs were significantly reduced to 38% of the control level (P<0.05), while the Oas1 mRNA level was not significantly different from that in the concurrent control ovaries (P>0.05, Fig. 5A) and PCNA proteins were obviously reduced (Fig. 5B). These observations demonstrate that under our experimental conditions, PCNA expression can be down-regulated efficiently and specifically by RNAi technology without invoking interferon response and cellular toxicity in fetal mouse ovaries.

### Down-regulation of PCNA by RNAi increases oocyte survival and thus primordial follicle assembly, but retards the development of primordial follicles into primary follicles

To understand the consequences of PCNA RNAi on the development of ovarian follicles, primordial and primary follicles were counted separately in sections from 18.5 dpc ovaries transfected with PCNA siRNAs or non-targeting siRNAs (control) for 96 hours (Figs. 5C, D). The ovaries transfected with PCNA siRNAs exhibited significantly more total (P<0.05) and primordial follicles (P<0.05) than the control ovaries. However, significantly fewer primary follicles were observed in PCNA RNAi ovaries than in the controls (P<0.01, Figs. 5C, D). Thus, these data indicate that knockdown of PCNA in mouse ovaries around follicle formation increases primordial follicle assembly, but delays the transition of primordial to primary follicles.

**Figure 6:**
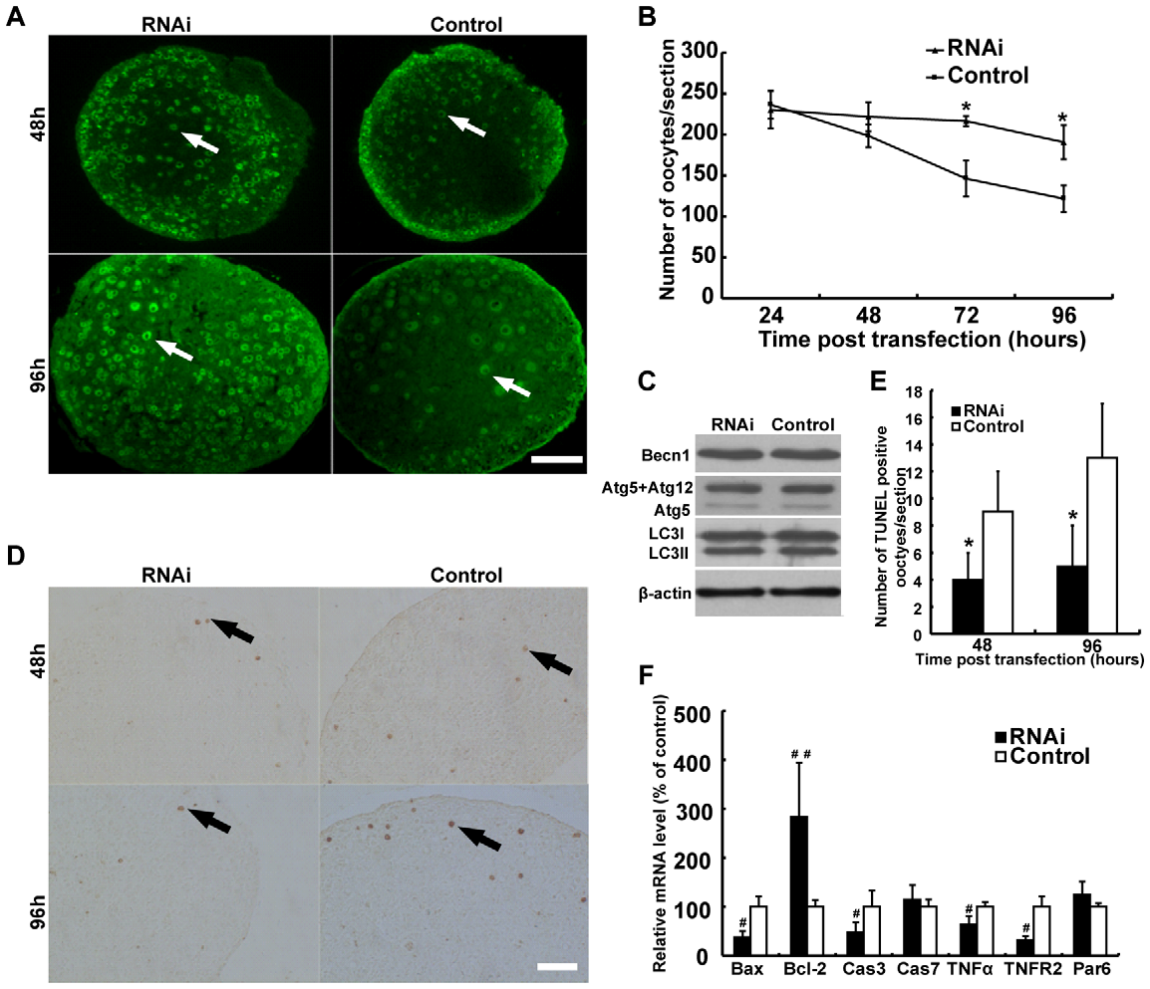
Down-regulation of PCNA increases the number of oocytes by alleviating the apoptosis of oocytes in fetal mouse ovaries. (**A**) Oocytes (arrow) were detected in sections of 18.5 dpc ovaries transfected with siRNAs targeting PCNA mRNA (RNAi) or nontargeting siRNAs (control) for 48 or 96 hours and immunohistochemically labeled with MVH antibodies (green). Bar: 100 µm. (**B**) The number of oocytes decreased gradually with time of transfection in PCNA RNAi or control ovaries, while the decrease in control ovaries was more dramatic than in PCNA RNAi ovaries. (**C**) Autophagy-related proteins were determined by Western blot in 18.5 dpc ovaries transfected with siRNAs against PCNA (RNAi) or nontargeting siRNAs (Control) for 96 hours. A representative image of three independent experiments with similar results was shown. (**D**) Apoptotic oocytes (arrow) were identified by the detection of DNA fragmentation with TUNEL agents in 18.5 dpc fetal mouse ovaries transfected with siRNAs targeting PCNA or nontargeting siRNAs for 48 and 96 hours. Bar: 50 µm. (**E**) Quantification of TUNEL positive oocytes in PCNA RNAi or control ovaries. (**F**) mRNA expression of apoptosis-related genes was quantified by real-time PCR in PCNA RNAi and control ovaries 96 hours after transfection. Each bar represents a mean±s.d. of 20 (B), 12 (D) and 16 (E) ovaries from three or five independent experiments in different animals. #: P<0.05, ##: P<0.01, unpaired t test, *: P<0.05, **: P<0.01, student's t-test, compared with concurrent controls.

An increase in the number of primordial follicles in neonatal mouse ovaries has been attributed to the increased number of oocytes during follicle formation [52]. We thus determined whether or not PCNA RNAi could promote oocyte survival. We counted oocytes in sections of cultured ovaries harvested every 24 hours after PCNA RNAi initiation compared with those in non-targeting siRNAs transfected control ovaries (Figs. 6A, B). A steady decrease in the number of oocytes was observed in both PCNA RNAi and control ovaries with the extension of culture (Fig. 6B). Strikingly, this decrease was much less severe in PCNA RNAi ovaries, with significantly more oocytes being observed at 72 and 96 hours after PCNA RNAi initiation when compared with the concurrent controls, respectively (P<0.05, Fig. 6B). Consistently, down-regulation by RNAi in 18.5 dpc mouse ovaries of Ing1b, a PCNA-binding protein in UV-induced apoptosis regulation [57], significantly increased the number of oocytes and primordial follicles than in control ovaries (Fig. S1B–E). Altogether, these results suggest that down-regulation of PCNA by RNAi in mouse ovaries around follicle assembly is able to alleviate oocyte loss, which could promote the formation of primordial follicles and delay the transition of primordial to primary follicles.

### The increase of oocytes in PCNA RNAi ovaries is due to decreased apoptosis of oocytes during follicle formation

Several possible mechanisms, including apoptosis [7], [8], [9], [10], [11], autophagic cell death [10], [12], [58], and oocyte extrusion from ovaries [12], have been proposed for oocyte loss, with the definitive elucidation of oocyte extrusion being impossible because of technologies available at present [12]. We thus analysed the expression of apoptosis- and autophagy-related genes after PCNA siRNAs transfection, to understand which mechanism(s) is/are responsible for the increased oocytes in mouse ovaries around primordial follicle formation after down-regulation of PCNA expression. All the autophagy markers tested, LC3I, LC3II, Atg5, and Becn1 [59] did not show any changes after PCNA RNAi (Fig. 6C). However, apoptotic oocytes, detected using the TUNEL assay, were observed in both PCNA RNAi and control ovaries at 48 and 96 hours after siRNA transfection (Fig. 6D, arrows). Significantly fewer apoptotic oocytes were noted in PCNA RNAi ovaries compared to controls (Fig. 6E). These results indicate that the increase in oocytes in PCNA RNAi ovaries results from decreased oocyte apoptosis rather than autophagy.

PCNA has been reported to participate in apoptosis of somatic cells [51],[57],[60]. Several genes from the Bcl-2 family [14], [61], [62], [63], TNF pathway [61], [64], caspase family [32], and PAR family [65] have been shown to be involved in the regulation of oocyte survival. We therefore investigated the expression of apoptosis-related genes after PCNA RNAi. As shown in Fig. 6F, the mRNA levels of known pro-apoptotic genes (Bax [Bcl-2 family], caspase-3 [caspase family], and TNFα and TNFR2 [TNF pathway], except caspase-7 [caspase family] and Par6 [PAR family]), decreased significantly in PCNA RNAi ovaries (P<0.05). The expression of Bcl-2 (Bcl-2 family), an anti-apoptotic gene, increased following PCNA RNAi (P<0.01). These results indicate that PCNA could regulate oocyte survival mediated by modulating the expression of apoptosis-associated genes.

### PCNA RNAi slightly decreases the proliferation of somatic cells that are not involved in primordial follicle formation

**Figure 7:**
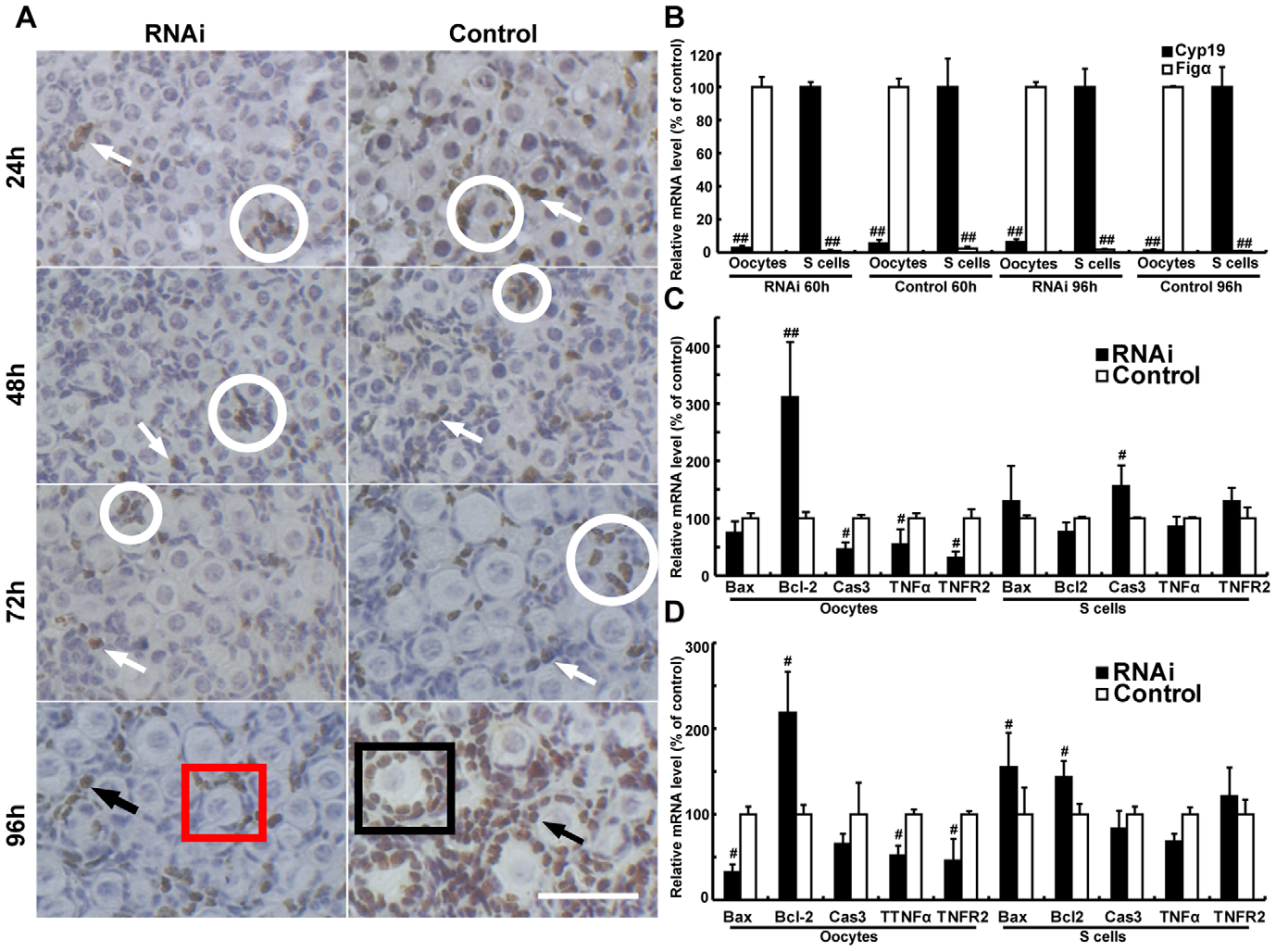
Down-regulation of PCNA increases oocyte survival, but decreases proliferation of somatic cells without effects on survival of somatic cells. (**A**) Proliferating somatic cells were labeled by an anti-BrdU monoclonal antibody in sections of control or PCNA RNAi ovaries, sampled at 24, 48, 72, and 96 hours after siRNA transfection, Respectively. BrdU was added into the culture medium 24 hours before ovary harvest. White arrows: BrdU-positive somatic cells; black arrows; BrdU positive granulosa cells; white circle: the areas showing that primordial follicles formed inactively; black box: a primary follicle showing that most granulosa cells are BrdU-positive; red box: a primary follicle showing a few BrdU-positive granulosa cells. Bar: 50 µm. (**B**) mRNA levels of Cyp19, a maker of somatic cells, and Figα, a marker of oocytes, were determined by real-time PCR in purified oocytes and somatic cells (S cells) in 18.5 dpc ovaries transfected with nontargeting siRNAs (Control) or siRNAs against PCNA for 60 and 96 hours. (**C** and **D**) mRNA expression of apoptosis-related genes was quantified by real-time PCR in purified oocytes and somatic cells at 60 (C) and 96 (D) hours after transfection. Each bar represents a mean±s.d. of 16 (B, C and D) ovaries from three or four independent experiments in different animals. #: P<0.05, ##: P<0.01, unpaired t test, compared with concurrent controls.

During primordial follicle formation, most pre-granulosa cells migrate and differentiate into squamous granulosa cells without proliferation [5], [7]. To determine whether or not altered proliferation of somatic cells could affect primordial follicle assembly and development, we measured cell proliferation in PCNA RNAi ovaries using BrdU incorporation as a marker of DNA replication. BrdU-positive somatic cells were observed in sections of cultured ovaries harvested every 24 hours after transfection initiation in both control and PCNA RNAi ovaries. In the first 72 hours after siRNA transfection, PCNA RNAi ovaries showed a slight decrease in the number of BrdU-positive somatic cells, when compared with the controls (Fig. 7A). However, because these BrdU-positive cells in both PCNA RNAi and control ovaries were cubic and mainly localized in the areas where the primordial follicle assembly was not active (Fig. 7A, white circle, white arrow), it is reasonable to suggest that these somatic cells were not participating in primordial follicle assembly. At 96 hours, significantly more primary follicles were observed in control ovaries and most granulosa cells in these follicles were BrdU-positive (Fig. 7A, red box). However, only a few primary follicles were observed in PCNA RNAi ovaries and these follicles usually had a small number of BrdU-positive granulosa cells (Fig. 7A). As expected, a majority of BrdU-positive somatic cells were cubic granulosa cells in primary follicles (Fig. 7A, black arrow). These observations were consistent with the expression of Ki-67 protein (Fig. S2C).

To further understand whether PCNA regulates primordial follicle assembly by promoting apoptosis of oocytes, without affecting somatic cells in mouse ovaries, oocytes, and somatic cells from cultured ovaries were isolated and used for detection of the expression of apoptosis-related genes. The purity of isolated oocytes and somatic cells were assayed by real-time PCR of Figα and Cyp19 [49], [50].

As expected, Cyp19 and Figα were expressed at very low levels in oocytes and somatic cells, respectively, demonstrating the high degree of purity (Fig. 7B). At 60 and 96 hours after PCNA RNAi, the pattern of expression of almost all apoptosis-related genes in isolated oocytes (Fig. 6F) was almost identical to that in PCNA RNAi ovaries, whereas only a slight and inconsistent expression of these genes was detected in somatic cells (Figs. 7C, D). These results indicate that PCNA RNAi mainly affected oocyte, but not somatic cell survival, in cultured ovaries and further supports the notion that proliferating somatic cells may not participate in the process of primordial follicle formation.

### Down-regulation of PCNA leads to more primordial follicles enclosed by fewer granulosa cells and delays the transition of primordial to primary follicles

**Figure 8:**
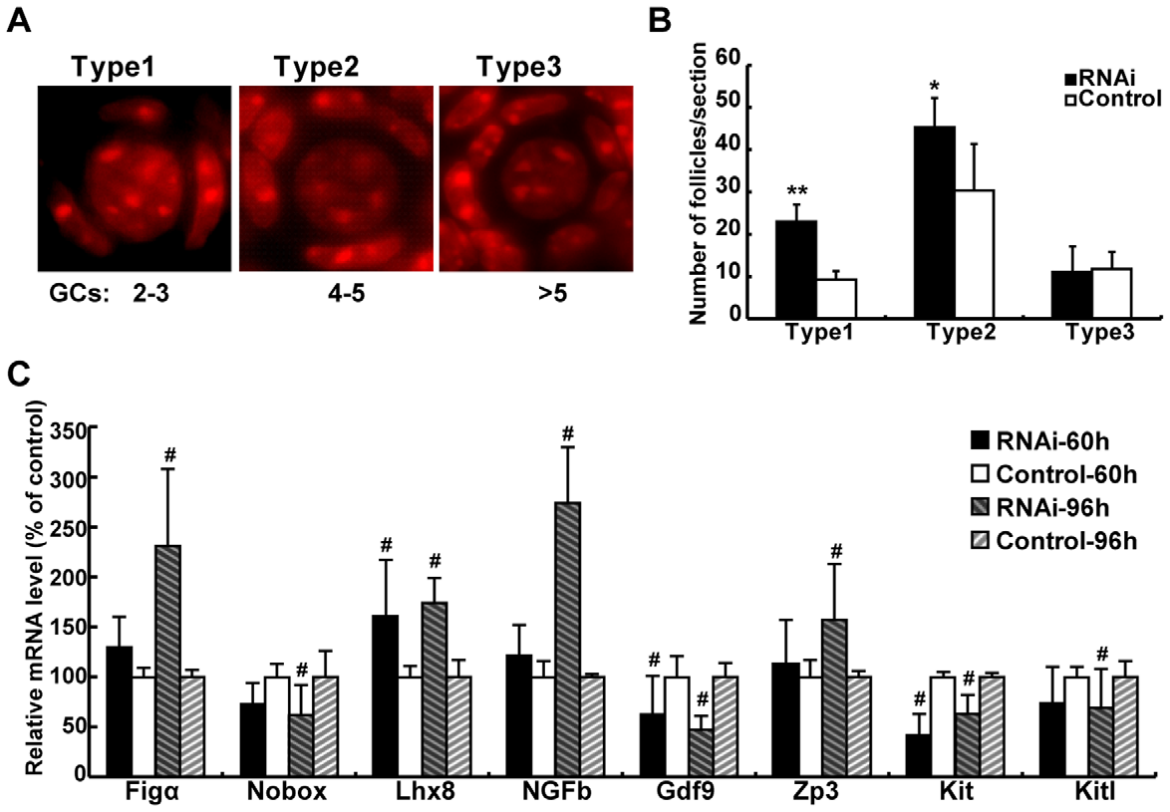
Down-regulation of PCNA increases the number of primordial follicles with fewer granulosa cells, and delays the transition of primordial follicles to primary follicles. (**A**) Based on the number of granulosa cells enclosing oocytes, the primordial follicles were grouped into three types, with type 1 having 2–3, type 2 4–5, and type 3 more than 5 granulosa cells. (**B**) Quantification of each type of primordial follicles in 18.5 dpc fetal mouse ovaries transfected with siRNAs targeting PCNA mRNA (RNAi) or nontargeting siRNAs (Control) for 96 hours. (**C**) The mRNA level of genes which regulated primordial follicle formation and transition of primordial to primary follicles were determined by real-time PCR in 18.5 dpc ovaries transfected with siRNAs against PCNA (RNAi) or nontargeting siRNAs (Control) for 60 and 96 hours. Each bar represents a mean±s.d. of 16 ovaries from three (B) or five (C) independent experiments in different animals. #: P<0.05, unpaired t-test, *: P<0.05, **: P<0.01, student's t-test, compared with concurrent controls.

In mouse ovaries, primordial follicle formation starts with two or three granulosa cells surrounding one oocyte, followed by more pre-granulosa cells migrating towards and enclosing the oocyte continuously [5]. To study the function of PCNA during primordial follicle formation more precisely, we classified primordial follicles arbitrarily into three categories, as described in the Materials and Methods (Fig. 7B). In PCNA RNAi ovaries, significantly more type 1 (P<0.01) and type 2 (P<0.05) primordial follicles were observed than in the controls, while the number of type 3 primordial follicles was similar to that in control ovaries (P>0.05, Figs. 7B, C). To detect whether or not the observed phenomenon that primordial follicles are enclosed by fewer granulosa cells after PCNA RNAi caused by the disruption of interactions between oocyte-granulosa cells or defects in oocytes, the expression of genes that play key roles in primordial follicle formation, e.g. Figα, Nobox, Lhx8, and NGFb [49], [66], [67], [68], were analyzed by real-time PCR at 60 and 96 hours after PCNA RNAi. With the increase in oocyte number and primordial follicle assembly, these genes increased consistently after PCNA RNAi (Fig. 8C). These results indicate that primordial follicles with fewer granulosa cells after PCNA RNAi did not have defects in the ability of oocytes to recruit granulosa cells.

Significantly fewer primary follicles were observed in PCNA RNAi ovaries than in the controls (Fig. 5D). We thus investigated the consequences of PCNA RNAi on the development of primordial follicles by detecting the expression of several genes (Gdf9, Kit, Kitl, ZP3, and Nobox) [66], [69], which were reported to be activated during development of primordial follicles after PCNA RNAi. Gdf9, Kit, Kitl, and Nobox were all decreased after PCNA RNAi for 96 hours, which is consistent with fewer primary follicles (Fig. 8C). To understand whether down-regulation of PCNA delays the transition of primordial to primary follicles, we analyzed the number of primordial and primary follicles in PCNA RNAi ovaries transfected for 6 and 8 days. Following RNAi for 6 and 8 days, primary follicles were still fewer in PCNA RNAi ovaries than in controls (Fig. S2A), however, the difference between PCNA RNAi and the control ovaries became small with the extension of culture time (Fig. S2B). Altogether, these results demonstrate that down-regulation of PCNA in mouse ovaries around primordial follicle formation delays the transition of primordial to primary follicles.

## Discussion

The expression of PCNA proteins during the development of fetal and neonatal mouse ovaries was carefully analyzed (Figs. 1, 2, 3). A typical V-shape expression pattern, i.e., decreasing expression in ovaries from 13.5–18.5 dpc and increasing expression in ovaries from 18.5 dpc to 5 dpp was observed by Western blot analysis (Fig. 2C). This finding is consistent with the expression of PCNA mRNA detected by real-time PCR analysis (Fig. 2D), and corroborated by the observation on the number of PCNA expressed oocytes and somatic cells in ovary sections (Figs. 2A, B). The decreasing PCNA expression in mouse ovaries aged from 13.5–18.5 dpc is further supported by a function analysis, showing that the expression of non-degradable PCNA arrested meiosis at the early pachytene stage in mouse testes [37] and oocytes in the pachytene stage were found mainly in 16.5 dpc to 1 dpp mouse ovaries (Fig. 3B). In contrast, the finding that all oocytes in adult mouse ovaries were labeled by PCNA antibody affirms the increasing PCNA expression from 18.5 dpc to 5 dpp [16], [18]. Interestingly, the differential expression of PCNA during the development of fetal and neonatal mouse ovaries was tightly correlated with the progression of meiotic prophase I and the development of follicles (Fig. 3). The frequency of PCNA-positive oocytes decreased in parallel with the decrease of oocytes in pre-leptotene, leptotene, and zygotene stages. This can be perfectly explained by the findings that the oocytes in these stages are undertaking DNA synthesis and repair [70], and PCNA plays a central role in DNA replication and repair as the processing factor of DNA polymerase [71]. This finding is also supported by the observation that oocytes at pachytene were not labeled by PCNA antibodies (Fig. 3A), and this variation in expression of PCNA was also found in fetal rat ovaries [22]. With the progression of meiotic prophase I, PCNA proteins were again detected in oocytes at diplotene, and the frequency of PCNA-positive oocytes increased rapidly with the accumulation of diplotene oocytes. The time of re-expression of PCNA in oocytes is coincident with the initiation of primordial follicle formation, followed by a rapid increase and maintenance of a high frequency of PCNA-positive oocytes during active primordial follicle formation. An increase in PCNA expression during primordial follicle formation has also been detected in mouse and rat ovaries by others [18], [20], [22].

The increased expression of PCNA in oocytes around the initiation of primordial follicle formation, and the observation that two-thirds of oocytes die during primordial follicle assembly [5], [7] suggest a role of PCNA in the regulation of oocyte fate, i.e., death or survival to form primordial follicles. After inhibition of PCNA expression by RNAi in ovaries during primordial follicle formation, surviving oocytes were significantly increased and apoptotic oocytes were correspondingly reduced (Figs. 6A–E). Overall, with the development of oocytes in meiotic prophase I, PCNA might play a central role in DNA replication and repair before pachytene stage, and then participate in regulation of apoptosis at diplotene stage during primordial follicle assembly. This was consistent with the increased expression of anti-apoptosis genes and suppression of pro-apoptotic genes (Figs. 6F, 8C). PCNA has been reported to participate actively in the regulation of apoptosis, either by promoting pro-apoptotic proteins, such as Ing1b or suppressing anti-apoptotic proteins, including Gadd45, MyD118, and CR6, in somatic cells [51], [57], [60]. Moreover, binding of Ing1b to PCNA has been shown to regulate the induction of apoptosis by UV irradiation [57], and Ing1b RNAi ovaries, like PCNA reduced ovaries, exhibited more oocytes and primordial follicles (Figs. 5D, 6B, Fig. S1B–E). These results not only indicate that Ing1b participates in apoptosis of oocytes, but also corroborate the observation that PCNA can regulate apoptosis of oocytes in fetal and neonatal mouse ovaries.

In mammals studied so far, germ cell loss is a striking feature of primordial follicle formation [5], [7]. In fetal and neonatal mouse ovaries, it was observed that approximately two-thirds of oocytes undergo cell death, whereas surviving oocytes develop into primordial follicles [5.7]. Several possible mechanisms, including apoptosis [7], [8], [9], [10], [11], autophagic cell death [10], [11], [12] and cell extrusion from ovaries [12], have been proposed for oocyte loss, with apoptosis of oocytes being the major mechanism revealed by almost all *in vivo* and *in vitro* studies by different groups [7], [8], [9], [13], [14]. This has been corroborated by observations in mouse models after deletion of apoptosis-regulating genes [13], [14]. Recently, autophagic oocyte death was observed by two groups when individual isolated oocytes but not intact ovaries were cultured *in vitro* [10],[11], even though, the apoptotic cell death was also found to be the major mechanism of oocyte death, and when newborn mouse ovaries were cultured in the absence of serum [12]. Recently, based on analysis of static images of mouse ovaries at various developmental points, Rodrigues *et al.* (2009) suggested that oocyte extrusion could result in loss of a significant fraction of oocytes during primordial follicle formation [12]. However, current technologies cannot definitively elucidate this process, as pointed out by the authors. Although multiple mechanisms might function simultaneously, our results, in agreement with many other studies, support the notion that apoptosis rather than autophagic cell death is a major mechanism for oocyte loss during primordial follicle assembly (Figs. 6C–F, 7B–D), and PCNA may participate actively in oocyte loss by regulating oocyte apoptosis.

The exact mechanism by which PCNA regulates apoptosis of oocytes during primordial follicle formation remains unknown. One possible explanation is that a surveillance mechanism may exist in ovaries around the time of primordial follicle formation and oocytes that do not satisfy the mechanism are eliminated through apoptosis. PCNA, perhaps in a manner analogous to P63, may serve as a component of this mechanism. Deletion of P63 in mouse ovaries protects oocytes from irradiation-induced apoptosis [72], [73]. Whether or not and how PCNA can protect oocytes with genetic damage from apoptosis remains to be tested. Nevertheless, our results provide strong evidence that PCNA plays an important role in apoptosis-mediated oocyte loss during primordial follicle formation in neonatal mouse ovaries.

Accompanying a decreased rate rate of apoptotic oocytes, more primordial follicles were observed in PCNA RNAi ovaries (Figs. 5C, D). This is consistent with earlier reports that deregulation in the expression of apoptosis-related genes from the caspase family, Bcl-2 family, and TNF pathway, which are responsible for oocyte survival, could lead to alteration of follicle assembly. For example, deletion of caspase-2 and Smpd-1 resulted in more surviving oocytes and promoted follicle formation in neonatal mouse ovaries [31], [32]. Knockout of Bcl-2 and Bcl-XL in mice decreased the viability of oocytes around the time of primordial follicle formation [13], [62]. Bax has also been shown to participate in the regulation of oocyte apoptosis during follicle assembly by several studies [61], [63], even though Greenfeld *et al.* reported that the increased oocytes and follicles in Bax knockout mice possibly resulted from the decreased apoptosis of PGCs prior to PGC colonization in the gonadal ridge [61]. TNFα and TNFR2 have also shown to regulate primordial follicle assembly through promoting oocyte apoptosis [64], [74]. Considering the decreasing rate of oocyte apoptosis and the increasing number of primordial follicles in PCNA RNAi mouse ovaries, we propose that PCNA promotes primordial follicles assembly by down-regulation of oocyte apoptosis during the perinatal stage.

Primordial follicles are formed by an individual oocyte enclosed within a single layer of squamous granulosa cells in neonatal mouse ovaries [7]. An oocyte arrested in the diplotene stage interacts with surrounding somatic cells (also called pre-granulosa cells) which progressively enclose the oocyte and ultimately transform into squamous granulosa cells around it to form a primordial follicle [7]. Pre-granulosa and squamous granulosa cells arrest in the G0 stage and undergo a growth phase until the transition from a primordial to a primary follicle, during which proliferation is exceedingly slow [75]. Thus, proliferating somatic cells are not likely to participate in primordial follicle assembly. Consistently, although somatic cell proliferation decreased slightly following PCNA RNAi, more primordial follicles were still formed (Fig. 7A, Fig. S2C). These results indicate that altered proliferation of somatic cells following PCNA RNAi in neonatal mouse ovaries did not affect primordial follicle assembly.

Following PCNA RNAi, primordial follicles with fewer granulosa cells (types 1 and 2 primordial follicles) significantly increased and those with more than five granulosa cells (type 3 primordial follicles) did not change (Figs. 8A, B). These observations may be explained partly by the following. (1) The number of surviving oocytes increased markedly, whereas the number of surviving pre-granulosa cells remained unchanged during primordial follicle assembly after PCNA RNAi (Figs. 6A–E, 7A, Fig. S2C). (2) In PCNA RNAi ovaries, some oocytes may maintain a normal capacity to recruit somatic cells and thus form type 3 primordial follicles, whereas others have a reduced capacity to recruit somatic cells and thus form types 1 and 2 primordial follicles. This suggestion needs to be tested experimentally.

Although more primordial follicles were observed after PCNA RNAi, the number of primary follicles decreased (Fig. 5D, Fig. S2A), accompanied by decreased expression of several key regulators of the transition from primordial to primary follicles (Fig. 8C). This inhibition of primordial follicle development could be explained by the decreased proliferation of granulosa cells after downregulation of PCNA (Fig. 7A, box). As the culture time increased, the difference in primary oocyte number between PCNA RNAi and control ovaries decreased, which can be attributed to the decreasing effect of PCNA siRNAs on proliferation of granulosa cells. In mice, developing follicles regulate the development of primordial follicles through secretion of TGF-β superfamily members, e.g. anti-Mullerian hormone, but these factors are not likely to regulate primordial follicle assembly in fetal and neonatal mouse ovaries according to the temporal development of follicles [76]. Therefore, in PCNA RNAi ovaries, although the number of primordial follicles increased, the development of these follicles was impaired.

In conclusion, the present study has demonstrated a dynamic expression of PCNA in germ cells and somatic cells during the development of fetal and neonatal mouse ovaries, which is tightly correlated with the progression of meiotic prophase I and assembly of primordial follicles. PCNA expression gradually decreased with meiosis progression from the pre-leptotene to pachytene stage, and steadily increased in diplotene oocytes around the time of primordial follicle formation when massive oocyte loss occurred. Suppressing the increase of PCNA by RNAi reduces apoptosis-mediated oocyte loss, and increases the number of primordial follicles. Although the exact mechanism by which PCNA RNAi promotes oocyte survival in ovaries around primordial follicle formation is unknown at the present time, our results indicate that PCNA plays a functional role during the well-known physiological process of germ cell loss around primordial follicle formation in mouse ovaries.

## Supporting Information

Figure S1 **The expression of PCNA is associated with the development of spermatocytes in adult testes, and down-regulation of Ing1b, a binding protein of PCNA in the regulation of the apoptosis induction, increases the number of oocytes and primordial follicles.** (**A**) An example of sections of paraffin-embedded testes from adult mice displayed differential staining of PCNA protein in spermatogonia (S), leptotene (L), and pachytene spermatocytes (P). Bar: 50 μm and 5 μm. (**B**) Follicles/oocytes were detected immunohistochemically in sections of 18.5 dpc ovaries transfected with nontargeting siRNAs (Control) or siRNAs (RNAi) against Ing1b for 96 hours. Bar: 25 μm. Quantification of oocytes (**C**) and follicles (**D**) in Ing1b RNAi and control ovaries. (**E**) Ing1b mRNA level was detected by real-time PCR in the Ing1b RNAi and control ovaries. Each bar represents a mean±s.d. of 20 (**B**), 12 (**C**) and 16 (**D**) ovaries from three or five independent experiments in different animals. #: P<0.05, unpaired t-test, *: P<0.05, student's t-test.
(TIF)

Figure S2 **Down-regulation of PCNA delays the transition of primordial to primary follicles and decreases proliferation of somatic cells.** The index of primary follicles (**A**) and the number of follicles (**B**) were determined in PCNA RNAi and control ovaries transfected for 6 and 8 days. (**C**) Proliferating somatic cells were labeled with an anti-Ki-67 monoclonal antibody in sections of control or PCNA RNAi ovaries, sampled at 24, 48, 72, and 96 hours after siRNA transfection of 18.5 dpc ovaries, respectively. Ki-67-positive cells were cubic and mainly localized in the areas where the primordial follicle assembly was not active during the first 72 hours (black arrow), and in primray follicles at 96 hours (white arrow) after transfection. Bar: 50 μm. Each bar represents a mean±s.d. of 12 ovaries from three independent experiments in different animals. *: P<0.05, student's t-test, compared with concurrent controls.
(TIF)

Table S1 Primers used for quantifying the levels of various transcripts present in mouse ovary.
(PDF)
