# Supplementary figures and images for: Proliferating Cell Nuclear Antigen (PCNA) Regulates Primordial Follicle Assembly by Promoting Apoptosis of Oocytes in Fetal and Neonatal Mouse Ovaries
Source: PLoS One. 2011 Jan 6;6(1):e16046. doi: 10.1371/journal.pone.0016046 (PMC3017099; doi:10.1371/journal.pone.0016046)

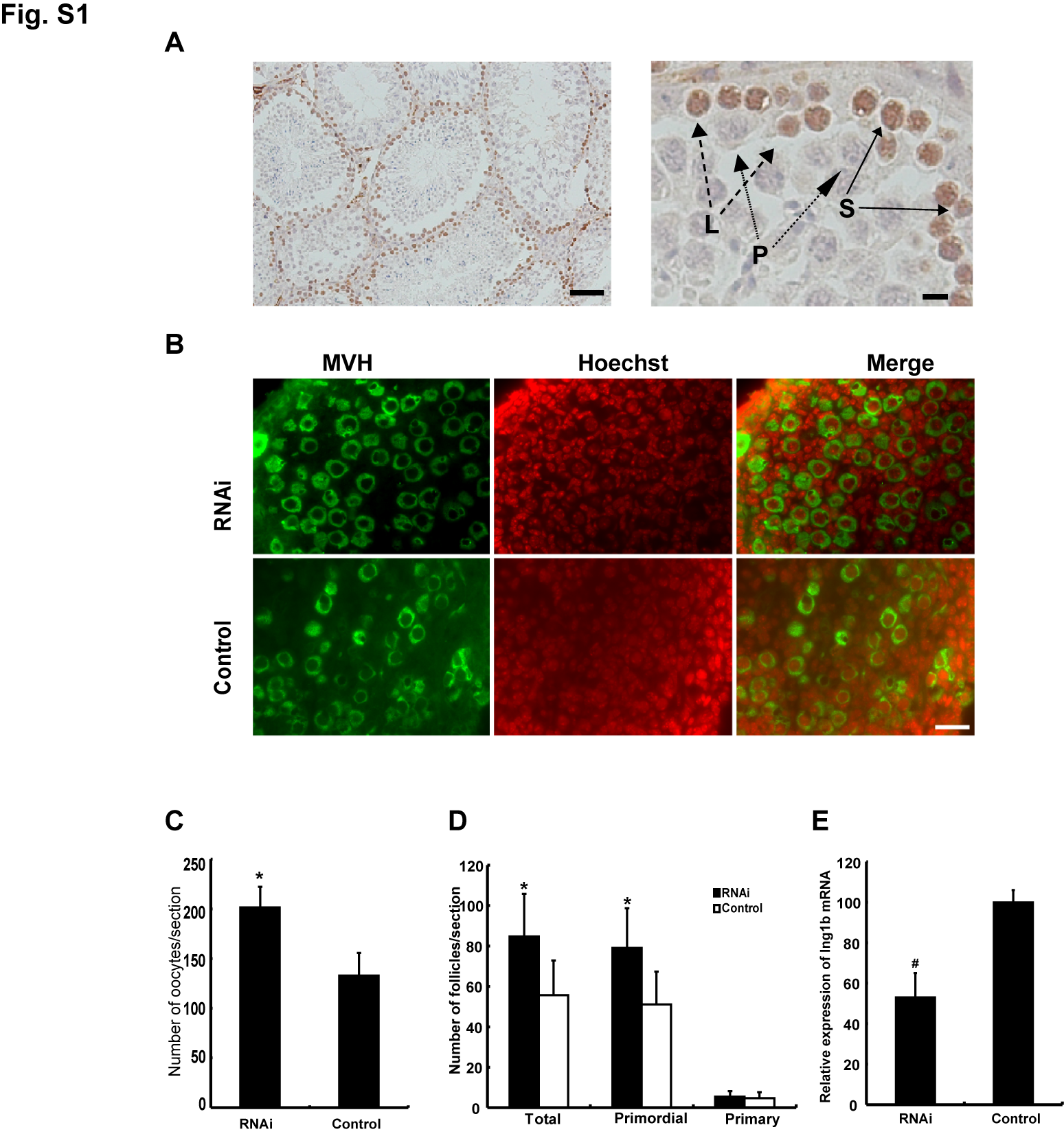

Supplement: Figure S1 — The expression of PCNA is associated with the development of spermatocytes in adult testes, and down-regulation of Ing1b, a binding protein of PCNA in the regulation of the apoptosis induction, increases the number of oocytes and primordial follicles. (A) An example of sections of paraffin-embedded testes from adult mice displayed differential staining of PCNA protein in spermatogonia (S), leptotene (L), and pachytene spermatocytes (P). Bar: 50 μm and 5 μm. (B) Follicles/oocytes were detected immunohistochemically in sections of 18.5 dpc ovaries transfected with nontargeting siRNAs (Control) or siRNAs (RNAi) against Ing1b for 96 hours. Bar: 25 μm. Quantification of oocytes (C) and follicles (D) in Ing1b RNAi and control ovaries. (E) Ing1b mRNA level was detected by real-time PCR in the Ing1b RNAi and control ovaries. Each bar represents a mean±s.d. of 20 (B), 12 (C) and 16 (D) ovaries from three or five independent experiments in different animals. #: P<0.05, unpaired t-test, *: P<0.05, student's t-test. (TIF) [file pone.0016046.s001.tif]

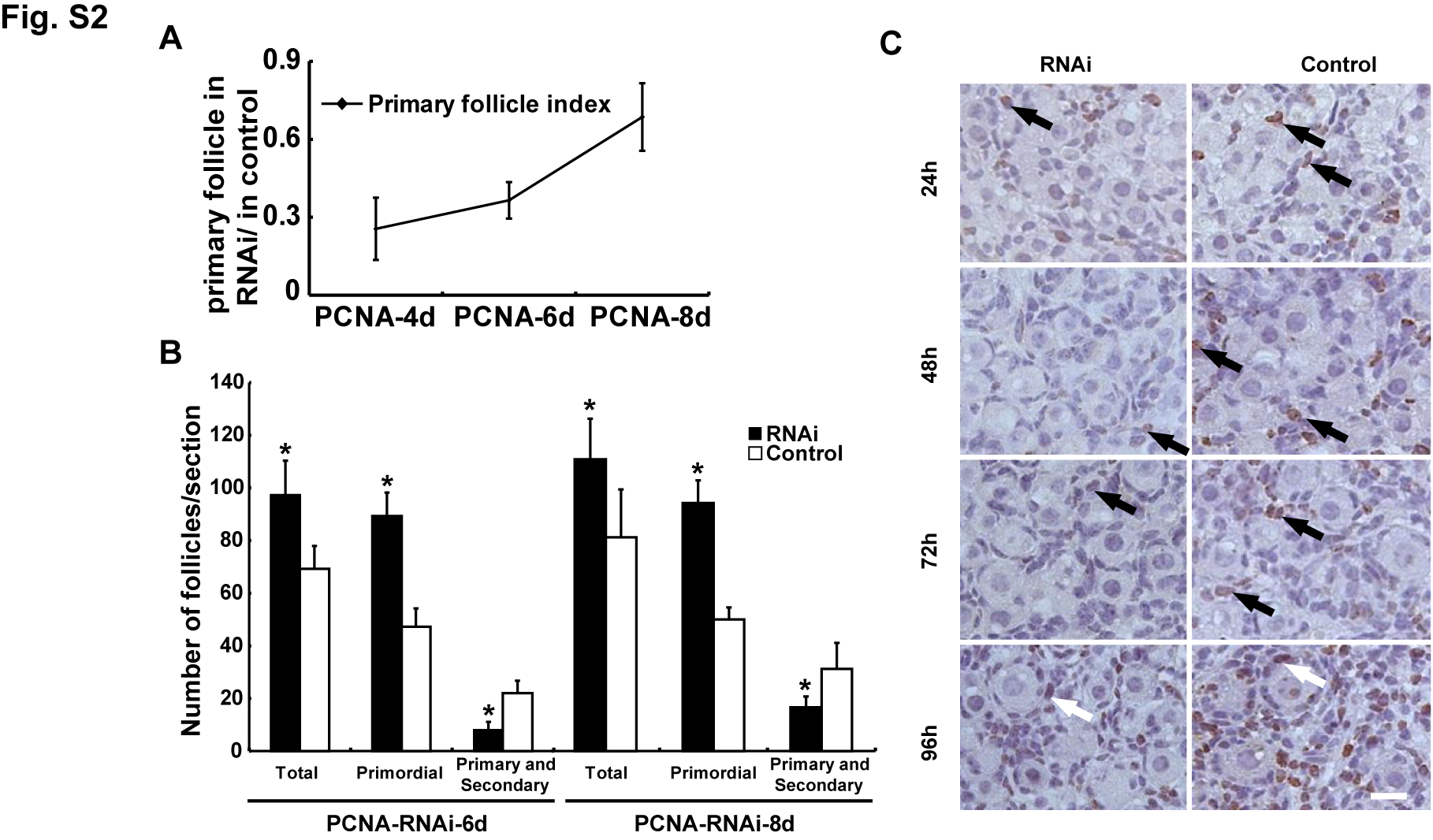

Supplement: Figure S2 — Down-regulation of PCNA delays the transition of primordial to primary follicles and decreases proliferation of somatic cells. The index of primary follicles (A) and the number of follicles (B) were determined in PCNA RNAi and control ovaries transfected for 6 and 8 days. (C) Proliferating somatic cells were labeled with an anti-Ki-67 monoclonal antibody in sections of control or PCNA RNAi ovaries, sampled at 24, 48, 72, and 96 hours after siRNA transfection of 18.5 dpc ovaries, respectively. Ki-67-positive cells were cubic and mainly localized in the areas where the primordial follicle assembly was not active during the first 72 hours (black arrow), and in primray follicles at 96 hours (white arrow) after transfection. Bar: 50 μm. Each bar represents a mean±s.d. of 12 ovaries from three independent experiments in different animals. *: P<0.05, student's t-test, compared with concurrent controls. (TIF) [file pone.0016046.s002.tif]
