# Supplementary material for: Proliferating Cell Nuclear Antigen (PCNA) Regulates Primordial Follicle Assembly by Promoting Apoptosis of Oocytes in Fetal and Neonatal Mouse Ovaries
Source: PLoS One. 2011 Jan 6;6(1):e16046. doi: 10.1371/journal.pone.0016046 (PMC3017099; doi:10.1371/journal.pone.0016046)
Supplement: Table S1 — Primers used for quantifying the levels of various transcripts present in mouse ovary. (PDF) [file pone.0016046.s003.pdf]

Table 1. Primers used for quantifying the levels of various transcripts present in mouse ovary

Genes	Forward primer (5'-3')	Reverse primer (5'-3')
PCNA	TAAAGAAGAGGAGGCGGTAA	TAAGTGTCCCATGTCAGCAA
GAPDH	GGTGAAGGTCGGTGTGAACG	CTCGCTCCTGGAAGATGGTG
Oas1	AGGAGCATCCCGGCCTCGAA	GCGGTGCCTTTGCCCTTGAC
BAX	CAGGATGCGTCCACCAAGAA	GCAAAGTAGAAGAGGGCAACCA
Bcl-2	TGGAGAGCGTCAACAGGGAGA	GCCAGGAGAAATCAAACAGAGGT
Caspase3	AGCAGCTTTGTGTGTGTGATTCTAA	AGTTTCGGCTTTCCAGTCAGAC
Par6	CTTGGCTATACGGATGCTCACGG	GGTCGTAGCAGGAGCCCTTTCTT
TNFR2	GCATCCTTACATCGTTGGGTTCA	TTTGTAGGCAGGAGGGCTTCTTT
TNF	AGAACTCCAGGCGGTGCCTATGT	GTGGGCTACAGGCTTGTCACTCG
Fxna	TATGAATGCCAGCGACCTGTATC	ACCGTGCTTCTTGAAGTCCTTGT
Lhx8	CAGTTCGCTCAGGACAACAA	CCTGCAGTTCTGAAACCACA
Kitl	GGCAAATCTTCCAAATGACTA	TACCATATCTCGTAGCCAACA
Gdf9	CTGATAGGCGAGGTGAGACC	GAGCCGGACGGTATTGTAGA
Ngfb	TTGATCGGCGTACAGGCAGAACC	GTCCACAGTGATGTTGCGGGTCT
Figα	ACAGAGCAGGAAGCCCAGTA	TGGGTAGCATTTCCCAAGAG
Nobox	CATGAAGGGGACCTGAAGAA	GGAAATCTCATGGCGTTTGT
Zp3	GAGCTTTTCGGCATTTCAAG	AGCTTATCGGGGATCTGGTT
Kit	ACGTGCAGCAACAGCAATG	GGCCAACCAGGAAAAGTTTG
Caspase7	GCCTCTGGGACTTTTGCTTTCA	GCTTGGCGTCAACTCCGTCT
